# Extracellular Vesicles and Damage-Associated Molecular Patterns: A Pandora’s Box in Health and Disease

**DOI:** 10.3389/fimmu.2020.601740

**Published:** 2020-11-16

**Authors:** Anna Picca, Flora Guerra, Riccardo Calvani, Hélio José Coelho-Júnior, Francesco Landi, Roberto Bernabei, Roberta Romano, Cecilia Bucci, Emanuele Marzetti

**Affiliations:** ^1^Fondazione Policlinico Universitario “Agostino Gemelli” IRCCS, Rome, Italy; ^2^Aging Research Center, Department of Neurobiology, Care Sciences and Society, Karolinska Institutet and Stockholm University, Stockholm, Sweden; ^3^Department of Biological and Environmental Sciences and Technologies, University of Salento, Lecce, Italy; ^4^Università Cattolica del Sacro Cuore, Rome, Italy

**Keywords:** Alzheimer’s disease, damage-associated molecular patterns, endo-lysosomal system, inflammation, innate immunity, mitochondrial-derived vesicles, Parkinson’s disease, quality control system

## Abstract

Sterile inflammation develops as part of an innate immunity response to molecules released upon tissue injury and collectively indicated as damage-associated molecular patterns (DAMPs). While coordinating the clearance of potential harmful stimuli, promotion of tissue repair, and restoration of tissue homeostasis, a hyper-activation of such an inflammatory response may be detrimental. The complex regulatory pathways modulating DAMPs generation and trafficking are actively investigated for their potential to provide relevant insights into physiological and pathological conditions. Abnormal circulating extracellular vesicles (EVs) stemming from altered endosomal-lysosomal system have also been reported in several age-related conditions, including cancer and neurodegeneration, and indicated as a promising route for therapeutic purposes. Along this pathway, mitochondria may dispose altered components to preserve organelle homeostasis. However, whether a common thread exists between DAMPs and EVs generation is yet to be clarified. A deeper understanding of the highly complex, dynamic, and variable intracellular and extracellular trafficking of DAMPs and EVs, including those of mitochondrial origin, is needed to unveil relevant pathogenic pathways and novel targets for drug development. Herein, we describe the mechanisms of generation of EVs and mitochondrial-derived vesicles along the endocytic pathway and discuss the involvement of the endosomal-lysosomal in cancer and neurodegeneration (i.e., Alzheimer’s and Parkinson’s disease).

## Introduction

Inflammation is part of the innate immunity response to pathogens or molecules released upon tissue injury, collectively indicated as damage-associated molecular patterns (DAMPs) (1). This non-specific first line of organismal defense is mounted upon binding of DAMPs to a set of pattern recognition receptors (PRRs), including Toll-like receptors (TLRs) and inflammasomes that sense DAMPs and elaborate an immune response (2, 3). Albeit DAMPs-triggered inflammation is protective towards harmful stimuli *via* the coordination of their clearance, promotion of tissue repair, and restoration of tissue homeostasis, an excessive inflammatory response in the setting of persistent stimuli may be detrimental. Indeed, if dysregulated or not timely resolved, inflammation contributes to the development of several disease conditions (e.g., autoimmune diseases, cardiovascular disease, neurodegeneration, and cancer) (4, 5). Hence, a hyper-resolution response aimed at limiting hyper-inflammation and triggered by DAMPs-activated/initialized innate immune cells is in place (6). This pro-resolving pathway is possibly mediated by suppressing/inhibiting inducible DAMPs (SAMPs) (6).

A large deal of research has been devoted to understanding the complex regulatory pathways involved in DAMPs production and trafficking. The endo-lysosomal system that includes a set of dynamic and inter-convertible intracellular compartments such as early-, recycling-, and late endosomes, and lysosomes is a major component of such response. Along with this, autophagosomes are autophagy executors that deliver intracellular contents to lysosomes (7). The fusion of endosomes and/or autophagosomes with lysosomes installs an acidic environment and enables cargo degradation for recycling unnecessary components into re-usable biological building blocks (e.g., carbohydrates, proteins, lipids, and nucleotides) within the cell (7). These events are accomplished *via* vesicle trafficking, protein sorting, and selective cargo degradation. In particular, two opposite sorting systems are in place: the endosomal sorting complex required for transport (ESCRT) that supports cargoes degradation and the retromer complex that allows specific retrograde cargo retrieval (7).

Mitochondria are highly interconnected organelles that form a dynamic network by contacting the endoplasmic reticulum (ER), lysosomes, and the actin cytoskeleton (8, 9). While inter-mitochondrial junctions allow mitochondrial membrane cristae remodeling between adjacent mitochondria (10), mitochondrial fusion enables the mixing of matrix and intermembrane space contents (11). Recently, an additional mechanism of mitochondrial interconnection based on tube-like protrusions (mitochondrial nanotunnels) has been described (9). Mitochondrial nanotunnels may be especially relevant in establishing connections between organelles immobilized within post-mitotic tissues (e.g., skeletal muscle, myocardium), in which fusion events are limited (9). Finally, Golgi-derived vesicles contribute to the maintenance of mitochondrial homeostasis through participating in mitochondrial dynamics (12).

Altered regulation of the endosomal-lysosomal system has been implicated in several age-related conditions, including cancer and neurodegeneration, and might therefore be targeted for therapeutic purposes (13). Remarkably, small extracellular vesicles (sEVs) isolated from primary fibroblasts of young humans have shown to ameliorate senescence biomarkers in cells obtained from old donors (14). A major task of the endosomal-lysosomal system is the disposal of dysfunctional, but not severely damaged mitochondria *via* a housekeeping process of mitochondrial quality control (MQC) (15). Herein, we provide an overview on vesicle trafficking along the endocytic pathway, the generation of exosome and mitochondrial-derived vesicles (MDVs), and discuss the involvement of the endosomal-lysosomal system in physiological and pathological conditions, including cancer and neurodegeneration [i.e., Alzheimer’s (AD) and Parkinson’s disease (PD)].

## Genesis of Endo-Lysosomal Vesicles

**Figure 1:**
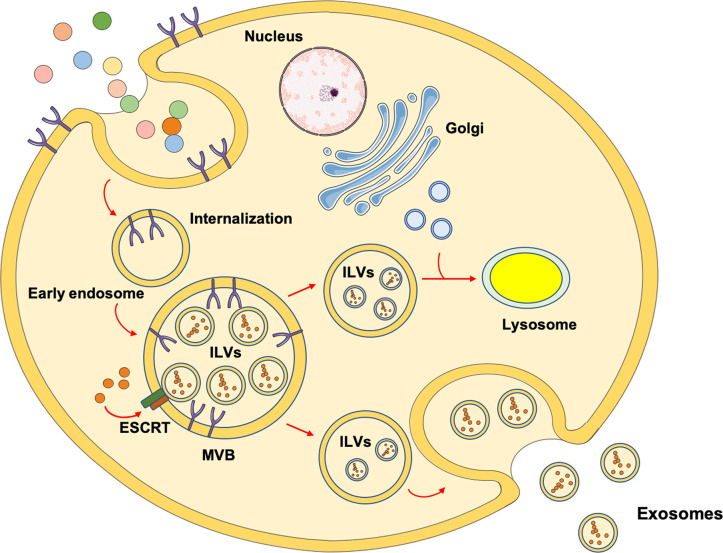
Schematic representation of the mechanisms involved in exosomes biogenesis. The most investigated mechanism through which exosomes are generated involves endocytosis after receptor/ligand binding at the cell’s membrane. After the ligand dissociates from its receptor, it is located into an early endosome. The receptor can either be recycled and relocated on the membrane surface or degraded into lysosomes. Through the activity of the endosomal sorting complex request for transport (ESCRT), the early endosome maturates into multivesicular bodies (MVBs) containing intraluminal vesicles (ILVs). Eventually, MVBs migrate toward the plasma membrane and fuse to release ILVs as exosomes. As an alternative route, MVBs can fuse with other MVBs or late endosomes and receive vesicles containing lysosomal enzymes from trans Golgi, evolving into lysosomes for degradative purposes.

Exosomes are EVs of endosomal origin with a diameter of 50-150 nm. The biogenesis of exosomes is associated with the generation and fate of multivesicular bodies (MVBs) (16). These organelles owe their name to the accumulation of intraluminal vesicles (ILVs) after inward budding of plasma membrane microdomains, fission, and release (16). ILVs have a small diameter (50-150 nm) and are identified as exosome precursors. As part of the endocytic trafficking, endosomal organelles undergo maturation and MVBs, moving from cell’s periphery to the center along microtubules, mature in late endosomes. For this reason, MVBs are considered to be newborn late endosomes derived from the maturation of early endosomes. However, according to an alternative model, MVBs are identified as intermediate transporters between early and late endosomes (17). Realistically, MVBs can follow two alternative directions: 1) toward fusion with other MVBs or late endosomes to undergo maturation and acidification, thus becoming lysosomes for cargo degradation or 2) toward the plasma membrane to fuse and release into the extracellular space ILVs, such as exosomes (16) (**Figure 1**).

Hence, the biogenesis of MVBs and exosomes is closely related. There are two different mechanisms that guide the origin of MVBs. In fact, they can originate *via* the sequential action of ESCRT or from endosomes containing lipid rafts (16, 18). The ESCRT system consists of five cytosolic complexes [i.e., ESCRT 0, I, II, III, and vacuolar protein sorting (VPS) 24] (19) and its role in exosome biogenesis has been proven by the identification of several ESCRT proteins in exosomes purified from different cell culture types or biological fluids. For this reason, ESCRT proteins are now used as exosomal markers (20).

ESCRT 0 recognizes a specific group of ubiquinated proteins on early endosomes referred to as phosphatidyl inositol monophosphate (PI3P) enriched domains. The recognition of ubiquitin and PI3P areas occurs through the interaction with the two subunits of the ESCRT 0 complex: HRS (hepatocyte growth factor regulated tyrosine kinase substrate) and STAM1/2 (signal transducing adaptor molecule 1/2). This is the first transition step from early endosomes to MVBs followed by the HRS-mediated recruitment of ESCTR I to endosomes (17, 21–23). ESCRT I in mammalian cells is a heterodimeric complex composed by tumor susceptibility gene 101 (TSG101), VPS28, VPS37A-D, and the ortholog of the yeast Mvb12 (22). In this step, the formation of stable vacuolar domains starts through TSG101 action carrying on the maturation of early endosome into MVBs (24). ESCRT I takes the place of ESCRT 0 and recruits ESCRT II that, in mammalian cells, is composed of ELL-associated protein of 30 kDa (EAP) 30, EAP20, and EAP45 (22). ESCRT III is a heterotetrameric complex composed by VPS20-CHromatin-Modifying Protein (CHMP) 6, Sucrose Non-Fermenting protein (SNF) 7-CHMP4, VPS24-CHMP3, and VPS2-CHMP2 subunits. ESCRT II recruits ESCRT III through the interaction between EAP20 and CHMP6, while CHMP-6 has been shown to regulate cargo sorting (25). ESCRT III has the role of recruiting deubiquitinating enzymes to remove ubiquitin residues from the protein with consequent complete invagination of the membrane and generation of ILVs. This is the last crucial step for the entry of cargoes into ILVs (22, 26). ESCRT III recruits accessory subunits, such as BRO1/ALIX (BCK1-like resistance to osmotic shock protein-1/apoptosis linked gene 2 interacting protein X) for cargo deubiquitination (27), and could also play a role in the fusion of MVBs with late endosomes (26, 28, 29). Finally, the interaction between ESCRT III and VPS4 allows the VPS4 ATPase activity to determine the final membrane budding, scission, and detachment of ESCRT subunit for recycling and cargo delivery (22). Thus, the whole process of ILV budding, cargo selection, membrane remodeling, and the incorporation of ILVs into MVBs is regulated by the ESCRT complex. However, only a few ESCRT components are necessary in this process, including HRS, TSG101, and STAM1 (ESCRT 0/I) (30). Indeed, the silencing of these proteins induces a decrease in exosome secretion, while an increase of exosome release is observed by inhibiting CHMP4C, VPS4B, VTA1 and ALIX (ESCRT III complex) (30). ALIX also interacts with several ESCRT proteins (e.g., TSG101 and CHMP4) and is involved in regulating protein composition/cargo loading, budding of ILVs, and MVB incorporation (31). Recent studies have also indicated that ALIX is crucial for the connection between syndecans and the ESCRT machinery through the binding of syntenins. Syntenins are soluble proteins acting as intracellular adaptors, *via* their PDZ domains that recruit syndecans. These latter are membrane proteins carrying heparan sulfate chains (HS) that are necessary to bind adhesion molecules and growth factors allowing them to interact with their receptors and assist in the endocytic process. This heterotrimeric complex is involved in endosomal budding and exosomes biogenesis (31, 32).

The exosome biogenesis can also follow an ESCRT-independent pathway. Indeed, even in the setting of simultaneous depletion of core ESCRT proteins, MVB and exosome biogenesis can still ensue *via* specific membrane lipid composition. Endosomes, which have domains enriched in cholesterol and sphingolipids, named lipid rafts, are able to curve inward and determine the formation of MVBs with the support of the pH gradient across the membrane (33). In this case, phospholipases mediate the synthesis of ceramides from sphingolipids and assure endosome membrane invaginations without ESCRT assistance. In fact, cone-shaped structures of ceramides, alone or associated with cholesterol, generate areas suitable for membrane deformation and ILV budding (34). The conversion of sphingomyelin in ceramide is catalyzed by neutral sphingomyelinases (SMases) which are enzymes located in the Golgi but also in the plasma membrane favoring exosomal biogenesis. Indeed, the inhibition of SMases reduces exosome secretion in specific cell types (35).

Originally identified in B lymphocytes and implicated in several cellular processes like cell fusion, cell migration and cell adhesion, the three tetraspanins CD9, CD81, and C63 are acknowledged as exosomal markers for their abundance in exosomes (36). These proteins generate the TEM domain (tetraspanin-enriched domain) and are composed by four transmembrane domains that interact with several other proteins, cholesterol, and gangliosides. Cargo sorting and formation of ILVs are mediated by the tetraspanins. Indeed, CD9 cooperates in the fusion of plasma membrane, while CD63 interacts with the PDZ syntenin domain (37).

The mechanisms through which MVBs move towards the plasma membrane for the release of exosomes instead of their fusing with lysosomes are presently unclear. Nevertheless, during the fusion of MVBs with the plasma membrane, the interaction between specific proteins and lipids determines exosome secretion, a process involving SNARE (soluble N-ethylmaleimide-sensitive fusion protein attachment protein receptors) proteins and small GTPases (38). Indeed, exosomes secretion is inhibited by overexpression of R-SNARE VAMP7 (vesicle-associated membrane protein 7), which induces enlargement of MVBs and their clustering at the cell’s periphery (39). The transport of MVBs towards the plasma membrane is regulated by microtubules and microfilaments such that the modulation of the expression of cortactin induces changes in the release of exosomes (40). Moreover, members of the Ras-related in brain (RAB) protein family, known for their role in endosomal trafficking, are also involved in exosome biogenesis and release. In this regard, several studies have shown a pivotal role for RAB27 and RAB35 in the docking of MVBs at the plasma membrane (41–43), while the silencing of RAB7A, the master regulator of late endocytic pathway, decreases syntenin-mediated exosome secretion (31, 44) or increases the release of CD9- and CD81-positive exosomes in cisplatin resistant cancer cells (45, 46).

DAMPs of different nature can be shuttled *via* EVs. Of note, mitochondria can also exploit this pathway for preserving organelle homeostasis. The mechanisms assisting in the generation of EVs from mitochondria are discussed in the next paragraph.

### Mitochondrial-Derived Vesicles

MDVs are generated by the selective incorporation of protein cargoes, including outer and inner membrane constituents, and matrix content. These vesicles have a uniform size (from 70 to 150 nm) and can follow two distinct fates: 1) they can fuse with MVBs and/or late endosomes for degradation (47) or extracellular secretion (13, 48); 2) they can be delivered to a subpopulation of peroxisomes (49).

Upon mitochondrial stress and isolation of mitochondria *in vitro*, it is possible to observe the formation of MDVs enriched in oxidized protein (50), revealing a mitochondrial stress-dependent selective cargo incorporation. An elegant work by Soubannier et al. (50) showed that MDVs carrying the outer membrane pore protein voltage-dependent anion channel (VDAC) are generated after the production of xanthine oxidase/xanthine-induced reactive oxygen species (ROS), while generation of ROS upon treatment with the complex III inhibitor antimycin A determines MDV formation without enrichment in VDAC, thus suggesting that MDVs can transport any oxidized cargo.

The protein kinase phosphatase and tensin homolog (PTEN)-induced putative kinase 1 (PINK1) and the cytosolic ubiquitin E3 ligase Parkin are required for the generation of MDVs targeted to the endocytic pathway and, finally, to the lysosomes (51). Both mutated in familial forms of Parkinson’s disease (52, 53), PINK1 and Parkin are known relevant factors in MQC and inducers of the mitophagic pathway. PINK1 is targeted to mitochondria but is normally degraded very rapidly (54–56). Indeed, during the import process at the site of mitochondria, a set of matrix processing peptidases and presenilins-associated rhomboid-like protein (PARL) cleave PINK1, thereby allowing its release from the mitochondrial import channel and subsequent cytosolic proteolytic degradation (56). However, in the setting of damaged mitochondria, the import machinery is inactivated thus determining the trapping of PINK1 within or near the import channel at the mitochondrial outer membrane (55). Here, PINK1, by exposing its kinase domain to the cytosol, induces Parkin phosphorylation. As a consequence, a stable recruitment of Parkin at the mitochondria and a Parkin-dependent ubiquitination of several proteins at the mitochondrial surface occur (57). Finally, a set of autophagic adaptor proteins recognize mitochondrial Parkin-ubiquitinated proteins and deliver damaged organelles to the autophagosome for subsequent disposal (57).

**Figure 2:**
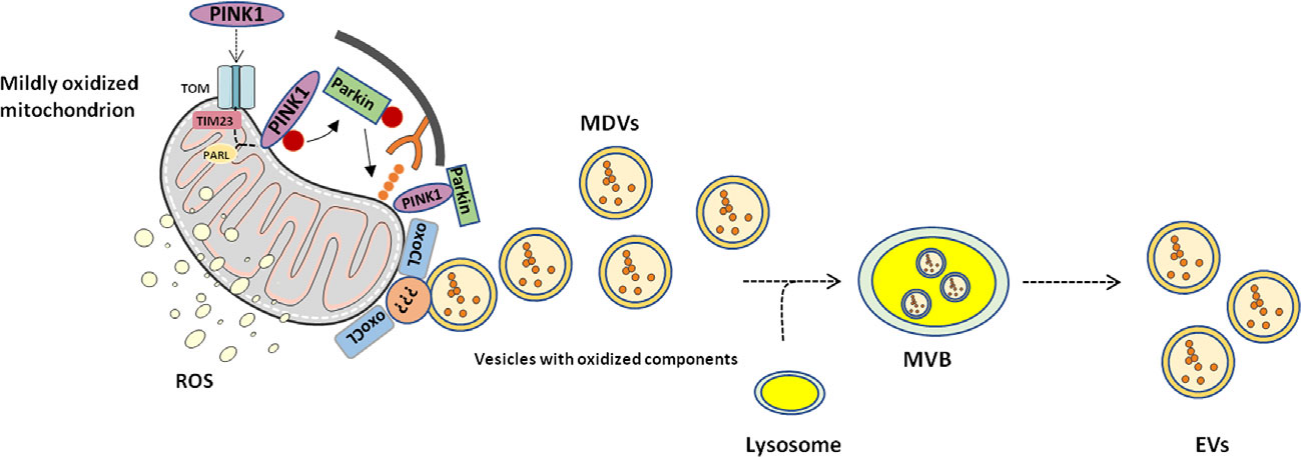
Proposed mechanism of mitochondrial-derived vesicle generation and release. Mitochondrial-derived vesicles (MDVs) may represent an additional level of mitochondrial quality control through which mildly damaged mitochondria are targeted and displaced. Phosphatase and tensin homolog-induced kinase 1 (PINK1) and Parkin prime damaged mitochondria for disposal. Membrane curvatures generated by oxidized cardiolipin (oxoCL) and other unknown proteins allow generation of MDVs that form multivesicular bodies (MVBs) within the endolysosomal system. Eventually, MVBs are extruded from the cell as extracellular vesicles (EVs). PARL, presenilin-associated rhomboid-like; ROS, reactive oxygen species; TIM23, translocase of inner mitochondrial membrane 23; TOM, translocase of the outer mitochondrial membrane.

Sugiura et al. (58) proposed a model in which they predicted a similar mechanism in PINK1- and Parkin-mediated MDV transport. The authors hypothesized that a local mitochondrial oxidative damage or complex assembly defects may induce protein aggregation at the mitochondrial import site that may clog the import process into the organelle. Along with this, the oxidation of phosphatidic acid and cardiolipin alters the membrane curvature which may support an early outward bending of the mitochondrial membrane, thus forming MDVs (59). Hence, a dual role for MDVs generation can be envisioned. On the one hand, MDVs can be considered as the first step of MQC, accomplished through the extrusion of damaged proteins as an attempt to avoid complete mitochondrial dysfunction. This would occur in the setting of mildly damaged organelles in which the autophagic pathway is not triggered (47, 51). On the other hand, severe mitochondrial dysfunction and uncoupling could induce a switch from local displacement of mitochondrial content to a complete arrest of PINK1 in all import channels, followed by the recruitment of autophagic mediators and degradation of the whole organelle (**Figure 2**).

Such a view supports the hypothesis of including the delivery of MDVs to lysosomes among MQC mechanisms. Indeed, cells perform MQCs *via* four different mechanisms: 1) degradation of unfolded and oxidized proteins within the mitochondrial matrix or intermembrane space by mitochondrial protease (60); 2) ubiquitination and delivery of mitochondrial outer membrane proteins to the cytosolic proteasome (61, 62); 3) activation of mitophagy to remove severely damaged mitochondria, whether linked to global protein misfolding or depolarization (63), and 4) generation and delivery of MDVs to lysosomes to protect the cell from premature mitophagy by removal of PINK1 and Parkin from each failing import channel.

## Damage-Associated Molecular Patterns and Sterile Inflammation

Chronic sterile inflammation ensues in several pathological conditions for which a common thread may reside into dysregulated EV trafficking. Therefore, a deeper understanding of the pathways generating EVs and triggering innate immunity may help clarify the events linking cellular dyshomeostasis with peripheral changes. The generation of MDVs orchestrated by mitochondrial-lysosomal crosstalk (64) is a strong candidate mechanism linking the two processes. Indeed, while operating as an housekeeping system in healthy mitochondria (16), in the setting of failing mitochondrial fidelity pathways, the clearance of dysfunctional organelles *via* MDVs may release noxious material with the potential of triggering inflammation (64). This response, mediated by the release of interferons (IFNs), pro-inflammatory cytokines, and chemokines, is part of innate immunity and starts with the recognition of an infectious agent (either viral or bacterial) that binds and activates membrane or cytoplasmic immune sentinel molecules termed PRRs [reviewed in (65)]. Of these, membrane-bound TLRs and the cytosolic retinoic-acid-inducible gene I (RIG-I)-like receptors (RLRs, RIG-I, and MDA5) are the best characterized in the setting of viral infections (66). Upon detection of double-stranded RNA produced during viral genome replication (67), TLR3 located in the endolysosomal compartment signals the binding *via* Toll-interleukin-1 receptor domain-containing adaptor inducing IFN-β (TRIF) and activates the IκB kinase (IKK) complex and the IKK-related kinases TRAF family member-associated NF-κB activator (TANK)-binding kinase 1 (TBK1) and IKKε. As a result of this activation, the translocation of nuclear factor-kappa B (NF-κB) and IFN-regulatory factors (IRFs) to the nucleus and their activation occur, thus inducing the production of type I and III IFNs together with a set of inflammatory chemokines including the regulated on activation normal T cell expressed and secreted (RANTES), and IFN-γ-inducible protein 10 (IP-10) (68–70). Viral RNAs can also be sensed in the cytoplasm by the RLRs, which signal *via* the mitochondrial antiviral signaling protein (MAVS) adaptor located at the mitochondrial outer membrane. Following the RLR-MAVS pathway, the activation of IKK and IKK-related kinases and, subsequently, NF-κB and IRFs occurs (71–73). Once induced, IFNs upregulate the expression of hundreds of IFN-stimulated genes (ISGs), ultimately installing an antiviral response that halts viral replication and spread (74). Along shared pathways, mitochondrial DAMPs can also trigger inflammation. In particular, mitochondrial DNA (mtDNA), due to its bacterial ancestry and its hypomethylated CpG motifs, is a potent trigger of innate immunity response involving the release of pro-inflammatory mediators installing an inflammatory milieu (75, 76). Indeed, mtDNA can interact with PRRs including TLRs, but also NOD-like receptors (NLRPs), and the cyclic GMP-AMP synthase–stimulator of interferon genes (cGAS–STING) systems (77, 78). The TLR pathway is engaged by mtDNA *via* its binding to TLR9 at the endolysosomal level, followed by the recruitment of the innate immune signal transduction adaptor myeloid differentiation primary response 88 (MyD88). The latter, by activating the mitogen-activated protein kinase, triggers inflammation *via* NF-κB signaling (79–81). Alternatively, mtDNA can ignite inflammation as part of the innate immunity response either *via* inflammasome or cGAS–STING system activation at the cytosolic level (82–86). The cGAS–STING DNA-sensing pathway operates *via* the TBK1/IRFs/IFNs pathways described above as part of inflammation mounted in the presence of viral infections (84–86). The activation of the STING pathway is also triggered as part of neutrophil activation and neutrophil extracellular trap (NET) formation, a specific cell death route characterized by the extrusion of chromatin-bound cytosolic content (87). NETs have been implicated in the pathogenesis of autoimmune disorders. In particular, NETs enriched in oxidized mtDNA stimulate a type I IFN response and have been implicated in lupus-like diseases (88). In systemic lupus erythematosus, mtDNA binding to the histone-like protein mitochondrial transcription factor A (TFAM) has shown to assist in rerouting oxidized mtDNA of neutrophils to lysosomes for degradation (89). Once extruded, TFAM-oxidized mtDNA complexes are powerful immune system activators (89). Similarly, the release of activated platelet-derived microparticles enriched with high-mobility group box 1 (HMGB1) protein has been described in systemic sclerosis (90). This DAMP molecule might contribute to vasculopathy and tissue fibrosis possibly *via* the presentation of HMGB1 to neutrophils to induce their activation and consequent endothelial damage (90).

Finally, the engagement of NLRP3, the best studied multi-subunit inflammasome system, elicits caspase-1 signaling and promotes caspase-1-dependent cleavage and activation of interleukin (IL) 1 and 18 *via* binding to adaptor molecules (91). This route of inflammation is particularly relevant to mitochondrial dysfunction since the synergistic activation of redox-sensitive inflammation and inflammasome reinforce inflammation (92). The molecular triggers of the inflammatory response *via* inflammasome are unclear. However, bacterial-like motifs of mtDNA are sensed by NLRs (93). Furthermore, NLRP3 is involved in facilitating the organization of the mitochondrial transition pore and assist in mtDNA release (94). A self-sustaining circle involving mitochondrial damage, ROS production, and consequent mtDNA damage/DAMPs release triggered by NLRP3 activators has been hypothesized (83). In particular, damaged/oxidized mtDNA/DAMPs are preferentially sensed and bound by NLRP3 (83).

Following the view of MQC failure as a source of MDVs/DAMPs, we will discuss in the next section the main literature supporting the involvement of mitophagy impairment and DAMPs release in the setting of cancer and two common neurodegenerative diseases (AD and PD).

## Implication of Extracellular Vesicles and Damage-Associated Molecular Patterns in Disease

### Cancer

**Figure 3:**
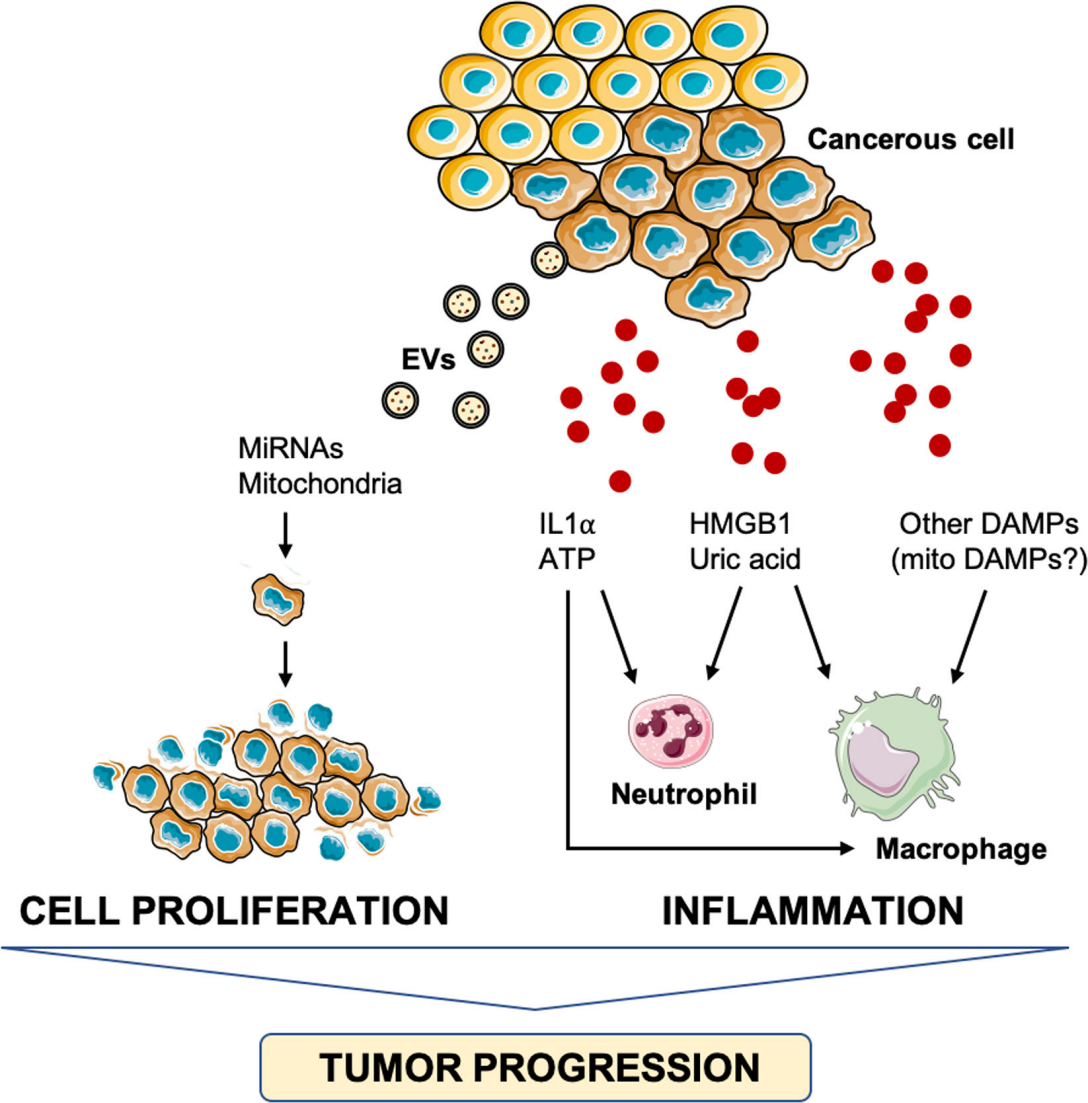
Schematic representation of the main pathways triggered by damage-associated molecular patterns and involved in tumor progression. ATP, adenosine triphosphate; DAMPs, damage-associated molecular patterns; HMGB1, high-mobility group box 1, IL1α, interleukin 1α; miRNA, micro RNA.

Although the involvement of DAMPs in cancer pathogenesis is debated, the installment of an inflammatory milieu is recognized as a factor favoring tumor progression (95, 96). In particular, increasing levels of pro-inflammatory mediators, including IFN-γ, IL1, IL6, lymphotoxin (LT)-β, tumor necrosis factor alpha (TNF-α), and transforming growth factor β, have been implicated in the promotion of carcinogenesis (95–97), for their potential role in modulating DAMPs expression and release (95, 98). Intracellular and extracellular DAMPs are, indeed, hallmarks of cancer that have been implicated in the early stages of carcinogenesis (95). While oxidative stress triggers the release of DAMPs in the extracellular space thus stimulating hyper-inflammation and immune injury, the loss of intracellular DAMPs, [i.e., HMGB1, histones, ATP, and DNA] induces genomic instability, epigenetic alterations, telomere attrition, reprogrammed metabolism, and impaired degradation (98). In the setting of such DAMPs-mediated pathogenic changes, cancer initiation and development are favored. Along with this, the release of ATP, IL1α, adenosine, and uric acid have also been implicated in carcinogenesis *via* induction of inflammation, immunosuppression, angiogenesis, and tumor cell proliferation (95) (**Figure 3**).

Strikingly, inflammatory pathways may also be activated by damaged mitochondrial constituents displaced within MDVs (65) that may trigger caspase-1 activation and secretion of pro-inflammatory cytokines (99). Interestingly, adaptive immunity responses are suppressed by PINK1 and Parkin that redirect MDVs toward lysosomal degradation to prevent endosomal loading with mitochondrial cargoes on major histocompatibility complex (MHC) class I molecules for antigen presentation purposes (48). Furthermore, the possibility that MDVs are used by cells as a homeostatic mechanism by horizontal mitochondrial transfer cannot be disregarded (100). Bone marrow mesenchymal stromal cells (BM-MSCs) eliminate damaged depolarized mitochondria through EVs and export them to neighbouring macrophages (101). Macrophages, in turn, recycle these MDVs to secrete exosomes which contain microRNAs (miRNAs) that inhibit TLR stimulation and induce macrophage tolerance to transferred damaged mitochondria (101). Moreover, cells with impaired mitochondria are able to transfer and take up fully-functional mitochondria displaced within MVDs to rescue aerobic respiration (102–105). A mitochondrial transfer was also shown between A549 mtDNA depleted (p^0^) lung cancer cell and BM-MSCs to rescue respiration in lung cancer cells lacking mtDNA-encoded subunits of the electron transport chain (ETC) (104). However, vesicles enriched in whole mitochondria or mitochondria void of envelops can also be released and serve as DAMPs in pathological conditions, including tissue injury and cancer (106). In particular, the release of mitochondria by damaged mesenchymal stem cells has been found to function as a danger signal to activate their rescue properties (107). The uptake of whole mitochondria by epidermal growth factor-activated human osteosarcoma cells *via* macropinocytosis has also been described (108).

Recent findings indicate that cancer cells can reprogram their energy metabolism to adapt and survive in unfavorable microenvironments *via* EVs (109). Indeed, an efficient mitochondrial respiration is required by cancer cells to maintain their tumorigenicity (110). Upon acquisition of mtDNA through EVs, estrogen receptor (ER)-positive breast cancer can evolve from hormonal therapy sensitive (HTS) to dormant (HTD) or resistant (HTR) with poorer outcome. EVs from patients with HTR disease contain full mitochondrial genome that might have been transferred to HTS/HTD cells to sustain oxidative phosphorylation, an exit from dormancy and the development of HTR disease (111). Additional findings show that EVs from melanoma, ovarian and breast cancer tissues contain mitochondrial membrane proteins and active mitochondrial enzymes that are not detected in healthy controls (112), thus corroborating the hypothesis that energy metabolism reprogramming in cancer cells may occur also *via* EVs.

Similar to HMGB1 and histones, miRNAs can also be releases in the extracellular space as DAMPs in cancer (113, 114). Recent work has shown an exosomes-dependent pathway to secrete miRNA in cancer cells (115). For instance, in pancreatic cancer cells, exosomes containing miR-212-3p are secreted and lead to decreased expression of MHC II in dendritic cells (DCs), thereby inducing immune tolerance (116). Another system used by cancer cells to escape their recognition by the immune system is based on PD-L1. This factor binds to the PD1 receptor on immune cells thereby inhibiting proliferation and survival of CD8+ cytotoxic T lymphocyte (117). A recent study has shown that exosomes derived from lung cancer express PD-L1 and this is implicated in immune escape and promotion of cancer growth (118). These mechanisms enable cancer cell survival, proliferation, and undisturbed dissemination into other bodily districts, even located at long distance from the primary neoplastic mass. Thus, exosomes are useful shuttles for cancer cells to elude the immune system’s response and achieve undisturbed survival and proliferation.

Moreover, cancer cells can also transfer miRNAs *via* exosomes to favor angiogenesis. These miRNAs of exosomal origin are ultimately DAMPs promoting cancer proliferation. Indeed, their secretion is induced under oxidative stress (119). An elegant work by Deng et al. (120) showed that gastric cancer cells released exosomes containing miR-155 to increase the expression of vascular endothelial growth factor (VEGF) and promote proliferation and tube formation of vascular cells. In further support to the role of exosomal miRNAs in promoting angiogenesis are findings showing a strong enhancement of angiogenesis and tumor growth in mice under the infusion of exosomes containing miR-155 (120).

Finally, DAMPs may also act as a suppressor of tumor progression by promoting immunogenic cell death. Under physiologic conditions, cell death linked to normal turnover is not immunogenic and does not activate PRRs, such as TLRs and NLRPs (121). In contrast, immunogenic cell death is essential for tumor suppression after chemotherapeutic treatments (122). Immunogenic and non-immunogenic cell death are characterized by different biochemical and metabolic events. In particular, during immunogenic cell death, antigens from dying cells are incorporated by DCs and presented bound to MHC to mount a T cell immune response. In this context, co-stimulatory signals and cytokines are required for differentiation of specific T cells (123). The preapoptotic exposure of calreticulin on the plasma membrane of dying cells promotes their uptake by DCs (124). Interestingly, the release of HMGB1 in the surroundings of dying cells (125) induces an increase in tumor antigen presentation and regulates the TLR4-dependent immune response (126). The role of the NLRP3 inflammasome is crucial for the immune response against dying tumor cells as it interacts with the adaptor molecule apoptosis-associated speck-like protein to induce caspase-1 activation (127). The caspase-1 pathway is involved in the production of proinflammatory cytokines (i.e., IL1β and IL18) which are essential to induce an immunogenic response (127). Notably, ATP released from dying tumor cells mediates immunogenic cell death *via* the activation of the NLRP3 inflammasome (128). Therefore, understanding the fine-tuning of DAMPs release may be crucial for unveiling new pathways that modulate tumor cell’s death vs. survival.

### Neurodegeneration

**Figure 4:**
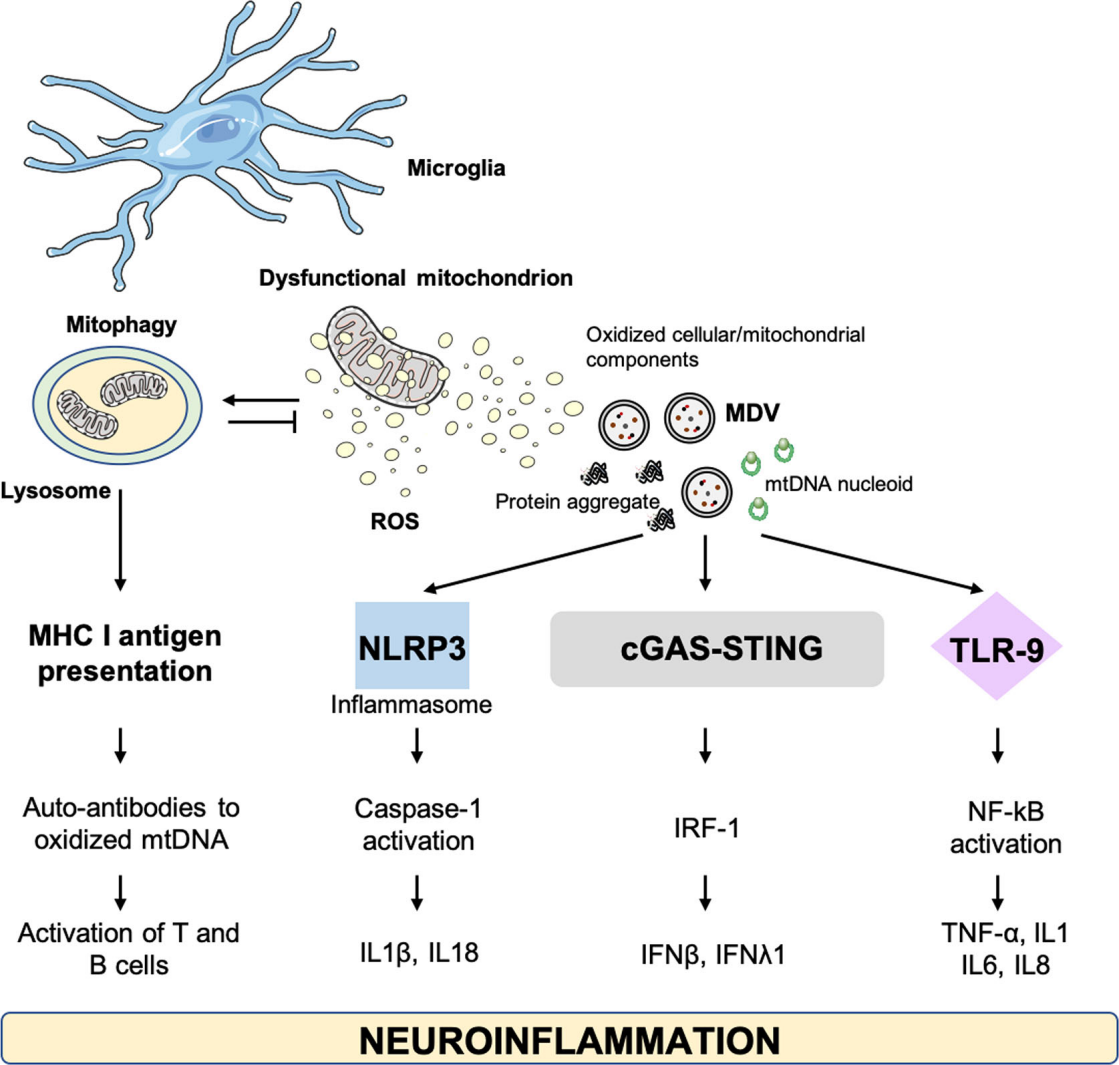
Cellular alterations and damage-associated molecular patterns involved in neuroinflammation. cGAS–STING, GMP-AMP synthase–stimulator of interferon genes; IFN, interferon; IL, interleukin; IRF-1, IFN-regulatory factor 1; MDV, mitochondrial derived vesicle; MHC, major histocompatibility complex; mtDNA, mitochondrial DNA; NLRP3, NOD-like receptor 3; NF-kB, nuclear factor kappa B; TLR, toll-like receptor; TNF-α, tumor necrosis factor alpha.

As a first line of defense against microbes, microglial cells of the central nervous system (CNS) preserve tissue homeostasis by clearing out damaged neurons and limiting the spread of infections. This macrophage population accomplishes these housekeeping activities by triggering inflammation *via* the release of cytokines and by instigating ROS production (129). However, upon prolonged stressors, a persistent microglia activation installs a pro-inflammatory and pro-oxidant environment that impinges on tissue homeostasis. A state of chronic, low-grade inflammation is observed during aging (i.e., inflamm-aging) which has been associated also with metabolic changes in microglia (130, 131). The age-related microglial and metabolic reshaping plays relevant roles in the context of AD and PD (132, 133). Indeed, neuroinflammation may represent a common thread in a large set of neurological disorders for which DAMPs of different origins, including mitochondrial, may support disease progression (134) (**Figure 4**).

#### Alzheimer’s Disease

AD is the most common age-associated dementia and is characterized by neuronal degeneration mainly in the *neocortex* and the *hippocampus* (12). The extracellular deposition of amyloid beta (A*β*) aggregates and intracellular neurofibrillary tangles are distinctive histopathological traits of AD (12). Amyloid plaque deposition instigates microglia activation which, in turn, promotes the development of a pro-inflammatory environment through the release of inflammatory cytokines, including IL1β, IL6 and TNF-α (135). This neuroinflammatory response may represent an inter- and intracellular signaling system between microglia and astrocytes aimed at clearing damaged neuronal components (136). Indeed, in the setting of inefficient intracellular quality control (137), the persistence of damaged components and hyper-inflammation may favor the generation and spread of A*β* peptides, thereby triggering neurotoxicity (135).

Dysregulation of the endo-lysosomal system contributes to the generation of amyloid plaques and AD pathogenesis. Indeed, A*β*42 aggregates, the most pathogenic A*β* peptides, have been detected in the soma of neurons at the level of lysosomes or lysosome-derived components (138). Furthermore, neurons from AD transgenic mice show enlarged and dysfunctional MVBs in the presence of A*β*42 accrual (139). As a consequence of MVB dysfunction, higher levels of the amyloid precursor protein (APP) are secreted extracellularly in this murine model (139). In the endosomal compartment is also located the activity of the β-site APP-cleaving-enzyme (BACE1), a hub for the intracellular trafficking of APP and a relevant contributor to amyloid plaque generation (140). Conversely, a retrograde transport of APP from endosomes to the trans Golgi network is in place to reduce A*β* production (141). Notably, an impairment in the retromer complex activity has been involved in AD pathogenesis (141).

Circulating levels of HMGB1 and the soluble form of the receptor for advanced glycation end products (RAGE) have been detected in the serum of AD patients. The concentration of these DAMPs correlate with the extent of A*β* deposition (142). Moreover, HMGB1 and thrombin proteins have been identified as pro-inflammatory mediators contributing to dysfunction of the blood-brain barrier (BBB) (142). Similarly, serum levels of the brain-derived protein S100B have been associated with the severity of the disease (143). The administration of the S100B inhibitor pentamidine was able to reduce the levels of S100B and RAGE and blunt A*β*-induced gliosis and neuroinflammation in a mouse model of AD (144).

Mitochondrial dysfunction and the ensuing oxidative stress have also been involved in the pathogenesis of AD. Indeed, a lower copy number and a higher levels of mtDNA heteroplasmy have been found post-mortem in brains of people with AD (145–147). In addition, oxidative damage to mitochondrial components has been described as an early event in AD, which suggests a role for oxidative stress in disease pathogenesis (148, 149). Interestingly, A*β* peptide aggregates and neurofibrillary tangles can impact mitochondrial function by binding to proteins of the mitochondrial import machinery (150). As a result, increased ROS production occurs (151). The mitochondrial localization of fragments of the E4 variant of apolipoprotein E, the main susceptibility gene for sporadic AD, has also been reported and associated with mitochondrial dysfunction and oxidative stress in hippocampal neurons (152, 153).

While primary mitochondrial deficits have been observed in AD, aberrant mitochondria can also result from defective quality control mechanisms, especially mitophagy. In particular, a vicious circle between defective mitophagy and mitochondrial dysfunction may be triggered A*β* and phosphorylated Tau (p-Tau), ultimately leading to neuronal disruption (154–156). Altered expression of the mitophagy receptor disrupted-in-schizophrenia 1 (DISC1) has been reported in AD patients, transgenic AD mice, and cultured cells treated with A*β* (157). DISC1 is a promoter of mitophagy that binds to microtubule-associated proteins 1A/1B light chain 3 (LC3) and protects synaptic plasticity from the toxicity of A*β* accrual (157). The positive effect exerted by the pharmacological restoration of mitophagy on cognitive dysfunction and A*β* proteinopathy in APP/PS1 mice highlights the central role of defective mitophagy in AD pathogenesis (158). Following pro-mitophagy pharmacological treatments, reduced levels of Tau phosphorylation and mitigation of inflammation induced by microglia activation have also been observed (154). As such, a link between neuronal bioenergetic failure resulting from defective MQC, inflammation, and neuronal loss can also be hypothesized in AD (92). Following mitophagy impairment, cGAS–STING-DNA-mediated inflammation has been described in neurodegeneration (159) and NLRP3-induced inflammation has been observed in AD [reviewed in (160)].

A defective mitophagy and the resulting accrual of dysfunctional mitochondria in AD may instigate the extrusion of damaged organellar components with consequent stimulation of innate immunity (77, 78). Mitochondrial DAMPs have been retrieved within circulating EVs in several age-related conditions, including neurodegeneration (161, 162). Whether this mechanism is relevant to AD is worth being explored.

#### Parkinson’s Disease

PD is the second most common age-related neurodegenerative disorder (163) and is characterized by a progressive degeneration of dopaminergic neurons of the *substantia nigra pars compacta* and dopamine depletion in the *striatum* (164). These histopathological and biochemical abnormalities underlie a set of motor (i.e., bradykinesia, postural inability, rigidity, and tremor) and non-motor signs and symptoms (e.g., constipation, depression, sleep disorders, cognitive dysfunction) (164).

Neuroinflammation is a noticeable feature of PD (165). In particular, the HMGB1-TLR4 axis seems to plays an important role. Higher serum levels of HMGB1 and TLR4 protein have been detected in PD patients and correlated with disease stage (166). Moreover, the administration of anti-HMGB1 monoclonal antibody in a rat model of PD was able to reduce inflammation by preserving the BBB and lowering IL1β and IL6 secretion (167). The chemokine fractalkine (CX3CL1), which is mainly expressed by neurons and serves as a modulator of microglial-neuronal communication, has been indicated as a possible biomarker for PD (168). Increased levels of the S100B protein were also detected in the *substantia nigra* and cerebrospinal fluid of persons with PD and in the ventral midbrain of a murine PD model treated with 1-methyl-4-phenyl-1,2,3,6-tetrahydropyridine (MPTP) (169). Notably, the ablation of S100B in the murine model was neuroprotective by reducing microgliosis and the expression of both RAGE and TNF-α (169). Noticeably, a systemic inflammatory signature, involving IL8, IL9, and macrophage inflammatory protein 1α and 1β, has been identified in older adults with PD (170).

A defective cellular quality control, manifested by deposition of aberrant α-synuclein in dopaminergic neurons, is acknowledged as an important mechanism underlying neurodegeneration in PD (171). The accumulation of α-synuclein at the mitochondrial complex I has been shown to impair its activity (171). Such an inhibitory function, together with mutations in genes encoding for the mitochondrial regulators Parkin, PINK1, and protein deglycase DJ-1 have been linked with enhanced ROS generation in PD (172, 173) and α-synuclein aggregation (174–177). Derangements in mtDNA homeostasis, including large deletions, have been also detected in neuronal cells of the *substantia nigra* of persons with PD (178–180). These observations indicate that mitochondrial dysfunction plays a major role in the pathogenesis of familial PD (181). On the other hand, PD in its sporadic form recapitulates all major hallmarks of aging (182). Indeed, MQC derangements and the generation of DAMPs have been indicated as a major contributors to the co-occurrence of mitochondrial dysfunction and neuroinflammation in PD (159, 183, 184). An innate immune response triggered by defective autophagy and impaired disposal of damaged mitochondria has been described in mice lacking PINK1 or parkin gene (PARK2) (159). Moreover, the activity of the mitophagy mediator Parkin mediates a mitophagic control over inflammation (48). In particular, Parkin regulates adaptive immunity *via* the presentation of mitochondrial antigens to endosomes for loading onto MHC class I molecules (48). Similarly, the intracellular trafficking regulator RAB7A exerts also a mitochondrial antigen presentation role by controlling the fusion of MDVs with late endosome for their subsequent degradation (48). The function of RAB7A as a mitochondrial antigen-presenting system in immune cells *via* MDV trafficking ensures that the process can be finalized in the absence of PINK1 or Parkin (48). Indeed, alterations in PINK1/Parkin expression and activity in PD result in MQC dysregulation and possibly neuroinflammation *via* mitochondrial antigen presentation by MDVs (48). Recent work by our group described the presence of mitochondrial DAMPs among circulating EVs in older adults with PD along with a specific inflammatory signature (162). In particular, higher serum concentrations of small EVs including exosomes of endosomal origin were identified in older adults with PD (162). However, lower levels of MDVs were retrieved in people with PD relative to non-PD controls (162). A lower secretion of MDVs in older adults with PD is in keeping with the hypothesis of intracellular accrual of dysfunctional mitochondrial secondary to engulfed MQC system (162). According to this view, MDV generation may serve as a housekeeping mechanism that complements MQC to preserve cell homeostasis (15). A link between mitochondrial damage and inflammatory and metabolic disarrangements in PD has also been proposed (184, 185); however, the molecular mechanisms linking these processes are missing. An involvement of the cGAS–STING-DNA driven inflammation in neurodegeneration following mitophagy impairment has been reported (159). Indeed, higher circulating levels of the pro-inflammatory cytokines IL6 and IFNβ have been detected in Pink and Parkin knockout mice challenged with exhaustive exercise (159). Notably, the deletion of STING or the administration of IFNα/β receptor-blocking antibody was able to blunt this response, thus suggesting that the accrual of dysfunctional mitochondria may trigger inflammation in people with PD (159). Among the ever-growing list of molecules linking mitochondrial dysfunction to systemic inflammation in PD, the fibroblast growth factor 21 (FGF21) has emerged as a relevant mediator (162). Indeed, FGF21 has been indicated as a “mitokine” for its association with impaired MQC in neurons of murine models of tauopathy and prion disease (186). Taken as a whole, these findings suggest that a deeper understanding on the intracellular and extracellular trafficking of DAMPs and vesicles, including those of mitochondrial origin, may be key to unveiling relevant pathogenetic pathways of PD and, hence, novel targets for drug development.

## Conclusion

Cells bearing DAMPs receptors sense and bind extracellular DAMPs as triggers of inflammation and fibrotic responses. Higher levels of circulating DAMPs have been identified during aging and related to inflamm-aging (3, 98, 187). A multicomponent senescence-associated secretory phenotype consisting of cytokines, chemokines (CXCLs), growth factors, and proteases has also been reported (188–191). While these secreted molecules contribute to preserving cell homeostasis in healthy tissues (192), the installment of an age-associated chronic secretory phenotype is a candidate pathway for the deployment of pathological hallmarks of aging, (e.g., inflamm-aging, tumorigenesis, loss of cell stemness). A core of circulating factors has been identified among plasma biomarkers of aging; however, their relationship with DAMPs is still unclear. The identification of circulating EVs stemming from altered regulation of the endosomal-lysosomal system in several age-related conditions, including cancer and neurodegeneration, holds hope for targeting this route for therapeutic purposes (13). Therefore, a deeper understanding of the complex, dynamic, intracellular and extracellular trafficking of DAMPs and vesicles, including those of mitochondrial origin, may be key to unveiling relevant pathogenic pathways and novel targets for drug development.

## Author Contributions

Conceptualization: AP, CB, EM, and FG. Writing (original draft preparation): AP, CB, EM, and FG. Writing (review and editing): RC, HC-J, and RR. Supervision: FL and RB. Funding acquisition: CB and RB. All authors contributed to the article and approved the submitted version.

## Funding

This work was supported by Innovative Medicine Initiative-Joint Undertaking (IMI-JU #115621), AIRC (Associazione Italiana per la Ricerca sul Cancro) Investigator grant 2016 #19068 to CB, Ministero dell’Istruzione, dell’Università e della Ricerca (MIUR) to Consorzio Interuniversitario Biotecnologie (DM 1049, 29/12/2018; CIB N. 112/19 to CB), intramural research grants from the Università Cattolica del Sacro Cuore (D3.2 2020), and the nonprofit research foundation “Centro Studi Achille e Linda Lorenzon”.

## Conflict of Interest

The authors declare that the research was conducted in the absence of any commercial or financial relationships that could be construed as a potential conflict of interest.
